# Psychiatric Disorder in Two Siblings with Hallervorden-Spatz Disease

**DOI:** 10.4306/pi.2009.6.3.226

**Published:** 2009-08-03

**Authors:** Young-Kyung Sunwoo, Jeong-Seop Lee, Won-Hyoung Kim, Yong-Bum Shin, Myung-Ji Lee, In-Hee Cho, Sun-Myeong Ock

**Affiliations:** 1 Department of Psychiatry, Inha University College of Medicine, Incheon, Korea.; 2 Department of Psychiatry, Gachon University of Medicine and Science, Incheon, Korea.; 3 Department of Family Medicine, College of Medicine, The Catholic University of Korea, St. Mary's Hospital, Seoul, Korea.

**Keywords:** Hallervorden-Spatz disease, Motor tics, Psychiatric symptoms

## Abstract

Hallervorden-Spatz disease (HSD) is a rare autosomal-recessive hereditary disorder characterized by the early onset of progressive movement alterations, including dystonia, rigidity, choreoathetosis, and mental deterioration. HSD is also associated with a variety of psychiatric symptoms, primarily depression and mental deterioration. However, psychosis has rarely been reported as a major symptom of HSD. We report two siblings who presented psychiatric symptoms as major clinical presentations, accompanied by ataxic and spastic gait, dysarthria, and typical neuroimaging findings of HSD. A 14-year-old girl presented complex motor tics, stereotypic behavior and anxiety symptoms. Her older brother, a 16-year-old boy, presented prominent auditory hallucinations, persecutory delusions and social withdrawal symptoms. Psychiatric symptoms were improved after atypical antipsychotic treatment. HSD is a rare disease but should be carefully considered in the diagnosis of patients with both motor disorder and various psychiatric symptoms.

## Introduction

Hallervorden-Spatz disease (HSD) is a rare autosomal-recessive hereditary disorder characterized by the early onset of progressive movement alterations, including dystonia, muscular rigidity, choreoathetosis, extrapyramidal symptoms, mental deterioration, dysarthria, and retinal degeneration.^1^,^2^

In order to diagnose HSD, four obligate, two or more corroborative and none of the exclusionary features should be presented.^2^,^3^ The obligate features are as follows: 1) onset during the first two decades of life, 2) progression of signs and symptoms, 3) evidence of extrapyramidal dysfunction and 4) hypodense areas on MRI involving the basal ganglia, particularly the globus pallidus and the substantia nigra. The corroborative features are as follows: 1) corticospinal tract involvement, 2) progressive deterioration of intelligence, 3) retinitis pigmentosa and/or optic atrophy, and 4) abnormal cytosomes in circulating lymphocytes and/or sea-blue histocytes in bone marrow. Exclusion criteria are as follows: 1) abnormal ceruloplasmin, 2) severe visual impairment and/or intractable seizure due to neuronal ceroid lipofuscinosis, 3) family history of Huntington's chorea and/or other autosomal-dominant movement disorders/caudate atrophy, 4) non-progressive course, and 5) absence of extrapyramidal signs.

Since HSD was first introduced by Hallervorden and Spatz in 1922, patients with this disease have been reported from all around the world. However, psychosis has rarely been reported as a major symptom of HSD.

A previous study reported the case of a 10-year-old boy who presented psychotic symptoms and was diagnosed as HSD. He was reported to have auditory hallucination, persecutory delusion, and anxiety about the "forthcoming destruction of the world".^4^

There have also been reports of patients who were diagnosed as HSD after presenting attention deficit hyperactivity disorder, stereotypies, and obsessive-compulsive behavior at young age.^5^,^6^ In addition, there were cases associated with various psychiatric symptoms such as depression, deterioration of personality, memory impairment, dementia, alcoholism, and conversion disorderlike symptoms.^1^,^7^ Tourette's syndrome had to be ruled out in this case because extrapyramidal symptoms during childhood are features of HSD. This paper reports a case of two siblings who were hospitalized in the psychiatric department and diagnosed with HSD due to the presence of complex motor tics, stereotypies and auditory hallucinations as major clinical symptoms. Patients and their parents were fully informed about the case report and a written informed consent was obtained.

## Case

### Case 1

A 14-year-old girl developed stereotypies and complex motor tics involving head turning to the left side and touching of the face after visiting her brother who was hospitalized in the psychiatric department. In October 2004, she became reticent and inarticulate, and her relationships with her classmates became disturbed. Three months later, she was hospitalized due to aggravation of stereotypies. Her perinatal period was uneventful, but delayed development of age-appropriate language was noted. No history of psychological or neurological disorders was found in any of her family members, except for her brother. On neurological examination, she could not stop rolling her eyes and turning her head to the left side. The Romberg sign was positive, and the heel-to-shin test was impaired. Ataxic gait, choreic movements, and dysarthria were observed.

Laboratory tests for serum ceruloplasmin, copper level in serum and urine, and liver function showed normal findings. Additionally, the Kayser-Fleischer ring was absent. In order to distinguish from Mitochondrial myopathy, encephalopathy, lactic acidosis, and stroke (MELAS), a lactic acid test was performed, which showed a slight increase of up to 3.1. The findings of laboratory investigations were unremarkable. In addition, urine homovanillic acid and serum neuron-specific enolase were normal.

Information processing ability to audio-visual stimuli was decreased to a moderate degree. Intellectual evaluation performed using the Korean version of Wechsler Intelligence Scale for Children (WISC) showed an intelligence quotient (IQ) of 88, which is within the lower limit of the average range. Aripiprazole (5 mg/day) was given for controlling persistent motor tic and stereotypies. Six days after the administration of medication there was a decrease in stereotypies and motor tics. Furthermore, she began to show some improvement in social withdrawal and anxiety.

**FIGURE 1:**
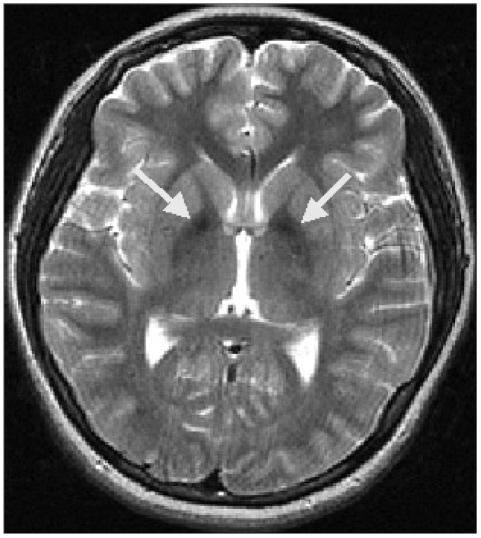
Magnetic resonance imaging. T2-weighted axial images showing areas of decreased signal intensity in the globus pallidus and the substantia nigra bilaterally.

A brain MRI demonstrated atrophy of the cerebellum and extrusion folia on T2-weighted sagittal images. T1-weighted axial images revealed hyperintense changes in the globus pallidus and the substantia nigra bilaterally, while T2-weighted axial images showed hypointensity in the same area (Figure 1). The patient was diagnosed with HSD based on her clinical features, brain MRI, and blood test results; however, the pantothenate kinase 2 gene (PANK2) gene mutation was negative in a genetic test that was performed in a general hospital in Seoul.

### Case 2

A 16-year-old boy, the older brother of the patient described in Case 1, had always been introspective and anxious. In October 2004, he exhibited leg weakness and an unsteady gait, and he fell down quite frequently. He was often left out by his classmates, and he began to spend a lot of time alone in his room. He was hospitalized in the psychiatric department of the general hospital.

On neurological examination, he was alert and his orientation was intact. He was anxious and irritable with an inappropriate affect. Auditory hallucination, loosening of association, persecutory delusion, delusion of being-controlled and the idea of reference were observed. There was no history of illness, and the findings of laboratory investigations were unremarkable. No abnormal results were found on tests of serum ceruloplasmin, copper levels in urine and serum, and liver function. In addition, the Kayser-Fleischer ring was not observed.

**FIGURE 2:**
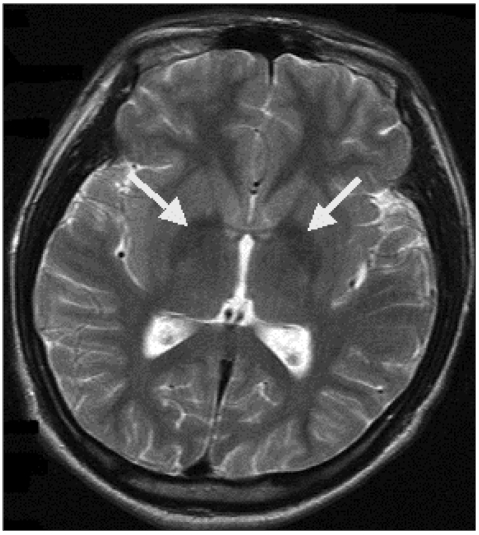
Magnetic resonance imaging. T2-weighted axial images showing areas of slightly decreased signal intensity in the globus pallidus bilaterally.

The Korean version of Wechsler Adult Intelligence Scale (WAIS) overall IQ was 71, which is considered to be a borderline score for mental retardation; however, his actual intellectual ability was inferred to be within the lower limit of the average range. Under the suspicion of schizophrenia, the patient was given risperidone (progressively increasing up to 6 mg/day). As the dose of medication increased, the patient began to develop dystonia and akathisia; nevertheless, he was treated as an outpatient, and the dose of medication was maintained. Brain MRI demonstrated areas of slightly decreased signal intensity in the globus pallidus on T2-weighted axial images, yet it was indistinguishable from normal status (Figure 2). The patient was diagnosed with HSD based on his family history and progressive features of dysarthria, intermittent dystonia, and abnormal gait. The PANK2 gene mutation was negative in genetic tests performed in a general hospital in Seoul.

## Discussion

Both patients in the two index cases described above were in their teenage years at the onset of HSD, and symptoms of extrapyramidal tract involvement, corticospinal tract involvement, mental retardation, family history, auditory hallucination, complex motor tic, stereotypies, depression, and anxiety were noted. In addition, MRI showed areas of decreased signal intensity in the globus pallidus on T2-weighted axial images, and the patients were therefore diagnosed as HSD.

The classification of HSD can be made using age at onset.^2^,^8^ 1) early-onset childhood types (before 10 years of age), rapidly progressive (classic) disease, 2) late-onset types (after 10 and before 18 years of age), slowly progressive (atypical) disease, and 3) adult types. The above two index cases are of late-onset type, so they are expected to progress slowly.

HSD is inherited as an autosomal recessive trait caused by a defect in the gene encoding PANK2, usually in the form of a missense mutation in all classic cases.^9^ Deficiency of PANK2 leads to the accumulation of cysteincontaining neurotoxic compounds that cause tissue damage and excessive iron accumulation in iron-rich brain regions.^8^ Recent study suggested that almost all of the classical cases have PANK2 mutations, with more prominent speech-related and psychiatric symptoms.^4^ Hayflick et al.^10^ found that only one-third of patients with atypical forms of the disease have mutations of the PANK2 gene. Our cases are considered to be atypical forms with no PANK2 gene mutation.

The basal ganglia is important in the pathogenesis of psychiatric symptoms in schizophrenia.^11^ Cognitive, affective, and motor symptoms of schizophrenia can be the result of abnormal output from the substantia nigra pars reticulata to the cingulate/orbitofrontal, dorsolateral prefrontal, and temporal cortical areas. Since the pathogenesis of HSD involves abnormal iron deposition in the globus pallidus and substantia nigra pars reticulata, lesions in those areas can cause symptoms of psychotic disorders, mood disorders, cognitive function impairment, stereotypies, etc.^4^,^11^

Although HSD is a rare disease that cannot be easily encountered in the psychiatric department, HSD should be considered in the diagnosis of patients with motor disorder along with various psychiatric symptoms. Doing so can be helpful in providing proper treatment by reducing the unnecessary use of medication and it will save time in making an exact diagnosis.
